# Supplementary material for: Using Cluster Theory to Calculate the Experimental Structure Factors of Antibody Solutions
Source: Mol Pharm. 2023 Apr 17;20(5):2738–53. doi: 10.1021/acs.molpharmaceut.3c00191 (PMC10155212; doi:10.1021/acs.molpharmaceut.3c00191)
Supplement: Supplementary file 1 — mp3c00191_si_001.pdf [file mp3c00191_si_001.pdf]

Using cluster theory to calculate the experimental structure factors of antibody solutions

Supplementary Information

Nicholas Skar-Gislinge,[†,‖] Fabrizio Camerin,[‡] Anna Stradner,[†,⊥] Emanuela Zaccarelli,[*,¶,§] and Peter Schurtenberger[*,†,⊥]

†*Physical Chemistry, Department of Chemistry, Lund University, SE-221 00 Lund, Sweden*

‡*Soft Condensed Matter, Debye Institute for Nanomaterials Science, Utrecht University, Princetonplein 5, 3584 CC Utrecht, The Netherlands*

¶*Institute for Complex Systems, National Research Council (ISC-CNR), Piazzale Aldo Moro 5, 00185 Rome, Italy*

§*Department of Physics, Sapienza University of Rome, Piazzale Aldo Moro 2, 00185 Rome, Italy*

‖*present address: Copenhagen Business School, Porcelaenshaven 18B, 2000 Frederiksberg, Denmark*

⊥*LINXS - Lund Institute of advanced Neutron and X-ray Science, Scheelevägen 19, SE-223 70 Lund, Sweden*

E-mail: emanuela.zaccarelli@cnr.it; peter.schurtenberger@fkem1.lu.se

Antibody excluded volume

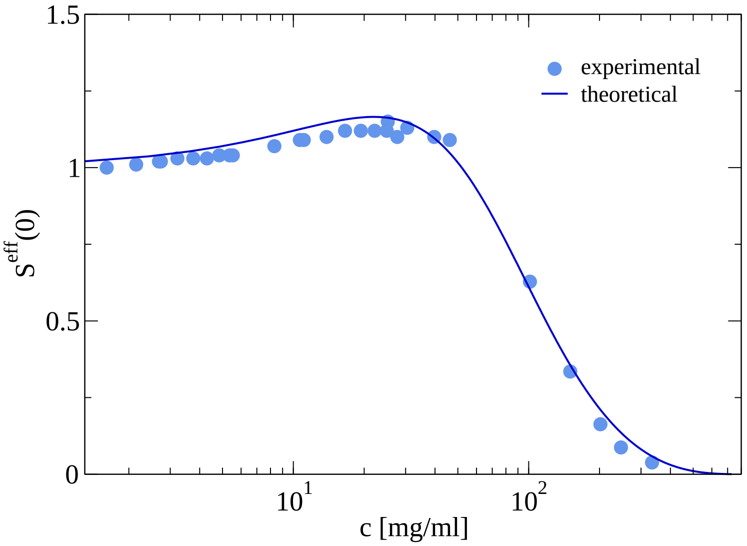

Supplementary Figure 1: Experimental and theoretical $S^{eff}(0)$ ($\sigma_{HS} = 2.95\sigma$, $T = 0.11$) as a function of concentration.

For developing a correct theoretical and computational model for antibodies, it is appropriate to check whether its excluded volume is correct with respect to experimental evidence. To this aim, we exploit the relationship between isothermal compressibility and the static structure factor $S(q = 0)$ via the number density ρ.

As explained in the main text, it is possible to map the coarse-grained Y-model to an effective hard sphere. To do this, as shown in Fig. S1, we first match the experimentally determined $S^{eff}(0)$ as a function of the concentration with the same curve obtained via Wertheim theory, allowing to identify the correct size of an effective hard sphere diameter σ_{HS}. Subsequently, to have a Y model that matches correctly the excluded volume, we compare the Y-simulation results of $S^{eff}(0)$ with the predictions of Carnahan-Starling obtained by using the σ_{HS} previously determined, as reported in Fig. S2. Here we compare the previously used 6-bead[1] and the newly chosen 9-bead model. We find that for the former case, the best agreement is obtained for $\sigma_{HS} = 2.7\sigma$ at low density and for $\sigma_{HS} = 2.54\sigma$ at higher concentrations. The latter value does not coincide with the one matching Wertheim theory results in the presence of patches.[1] Instead, for the 9-bead model, we find that the

isothermal compressibility actually reproduces the correct behavior with the expected HS size, namely $\sigma_{HS} = 2.95\sigma$. Importantly, the 9-bead model is able to capture the hard sphere compressibility in the whole experimental concentration range, thus appearing to be a superior model than the 6-bead model. Therefore, despite a slight increase in the number of beads, in the main text we focus on this model to provide a comparison for the experimental structure factors.

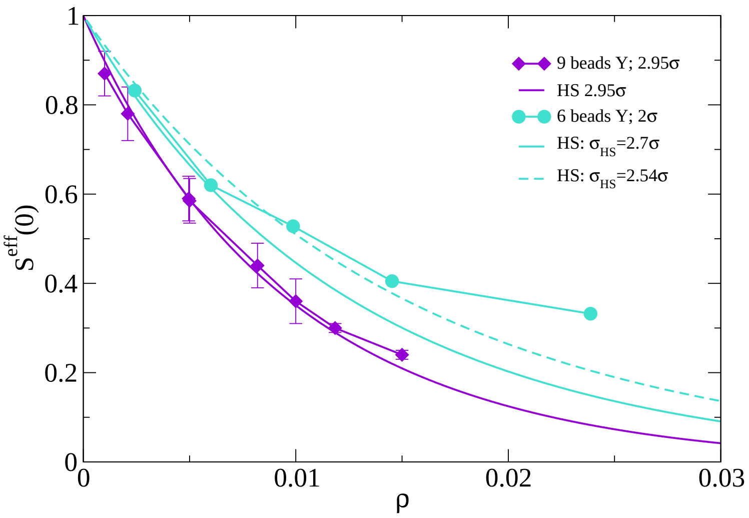

Supplementary Figure 2: $S^{eff}(0)$ as a function of the number density ρ for 9 and 6-beads Y models. The corresponding Carnahan-Starling (CS) results are also reported.

Simulation snapshots

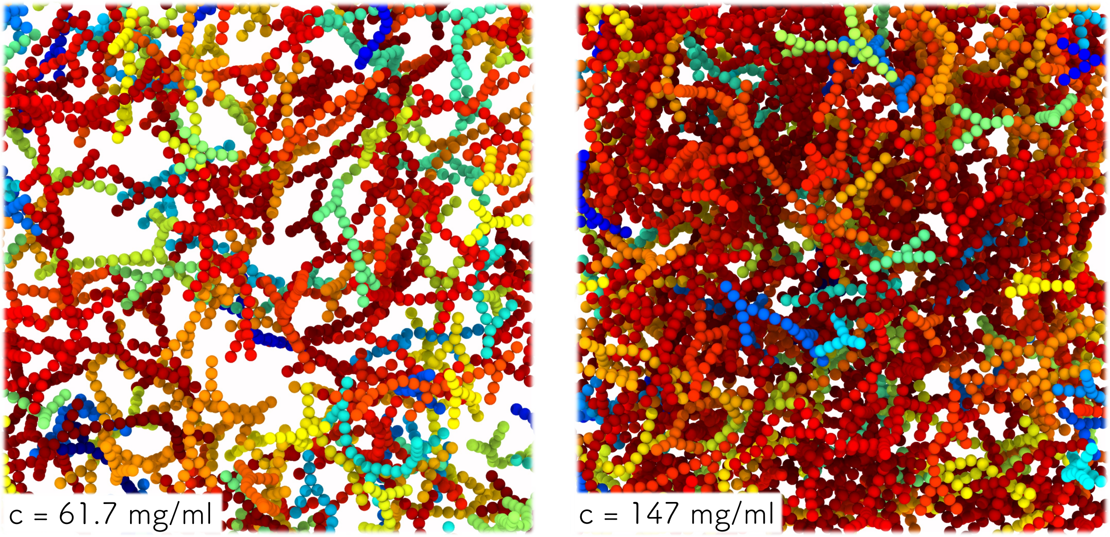

Supplementary Figure 3: Simulation snapshots of the antibody system for $c = 61.7$ and 147 mg/ml. Clusters are highlighted with different colors. Antibodies belonging to the same cluster are colored alike.

In Figure S3, we report two simulation snapshots for two different concentrations analyzed in the main text, namely $c = 61.7$ mg/ml and 147 mg/ml. Individual clusters of different sizes are reported in the main text.

References

(1) Skar-Gislinge, N.; Ronti, M.; Garting, T.; Rischel, C.; Schurtenberger, P.; Zaccarelli, E.; Stradner, A. A Colloid Approach to Self-Assembling Antibodies. *Molecular Pharmaceutics* **2019**, *16*, 2394–2404.
